# Female Nurses in Male Asylum Wards

**Published:** 1915-08-14

**Authors:** 


### Female Nurses in Male Asylum Wards.

The following manifesto, the contentions of which are opposed to the experience and results of the system obtained at Morningside and other great asylums, has been issued by the Executive Council of the National Asylum-Workers' Union :

The Executive Council of the National Asylum-Workers' Union view with serious concern the adoption by many asylum authorities, with the approval of the Board of Control, of a policy which has for its object the partial substitution of female nurses for male attendants in the care and charge of insane male patients in public asylums, and desire to record their unanimous condemnation of a practice which will, they are convinced, if persisted in, prove physically and morally detrimental to the nurses and injurious to the patients, besides impairing the efficiency of internal asylum administration, and possibly prejudicing the positions of those of our male members now serving with the Colours.

They firmly believe, having each personal and varied experience of the duties attaching to the treatment of male lunatics of all classes, that the work is of a nature entirely unfitted for women to perform; involving degrading duties repellent to all the finer instincts of chaste womanhood, and accompanied by a danger understandable only by those actively, intimately, and constantly associated with asylum work. From the point of view of the patients' welfare they deprecate the bringing of male patients, often morally perverted, into close association with persons of the opposite sex, and they give warning that the substitution of the less effective control of female nurses over male patients for the essentially firm supervision of male attendants is likely to give rise to an insubordinate and mutinous spirit among the least tractable inmates that may gravely endanger the safety of both patients and staff.

It is the opinion of this Executive Council that neither asylum authorities nor the Board of Control have given this subject the consideration which its importance demands, but have, on the contrary, prematurely embarked upon an experiment for which there is no justification; the alleged shortage of men for asylum duties being more apparent than real, as is proved by the facility with which other asylums, adjacent to those now employing females in male wards, are able to procure male substitutes for those of their staffs who have enlisted in the Army.

While it is quite possible to conceive that circumstances may arise before the conclusion of hostilities necessitating a departure from existing practices, none have yet arisen sufficient to warrant the substitution of female labour in a sphere where the employment of young women has hitherto, for the soundest medical reasons, been debarred; and in requiring their female employees, frequently against their wills, to undertake such duties, the asylum authorities concerned are exercising an unfair advantage and exerting undue pressure, having regard to the nurses' contract of service, which binds them, under penalty of dismissal, "to perform any duty allotted, although not of a nature which they usually perform."

With the object of combating a pernicious and dangerous innovation, the Executive Council are prepared to accord the fullest support to any protest made by female members of this Union, provided such protest is made in accordance with the procedure laid down in the Rules of the Union, and after approval by the Executive Council; and all branches affected are requested to offer every facility to our female members to avail themselves of the effective backing of the Union in any steps they may determine upon. In the last resort the defeat of the experiment depends upon the good sense and womanly delicacy of the nurses themselves, and upon the amount of opposition which their finer feelings will engender against the scheme.

Only in the event of it being conclusively proved that male labour is quite unprocurable on any reasonable terms will the Executive Council feel justified in suspending active opposition to the employment of female nurses in male Asylum wards, and then only on the understanding that equal pay, emoluments, &c., to both sexes.

